# Quantum valley Hall effect in wide-gap semiconductor SiC monolayer

**DOI:** 10.1038/s41598-020-61906-2

**Published:** 2020-03-19

**Authors:** Kyu Won Lee, Cheol Eui Lee

**Affiliations:** Department of Physics, Korea University, Seoul, 02841 Republic of Korea

**Keywords:** Quantum Hall, Topological matter

## Abstract

We have investigated the valley Chern number and gapless edge states in wide-gap semiconductor SiC and BN monolayers by using the density functional theory calculations. We found that while SiC monolayer has a non-quantized valley Chern number due to a partial mixing of the Berry curvature peaks pertaining to the opposite valleys, there exist topologically protected gapless edge states within the bulk gap, leading to a quantum valley Hall effect. Doping of the opposite charge carriers causes a backscattering-free valley current flowing on the opposite edge, which can be used for experimental confirmation and application at room temperature. BN monolayer, on the other hand, was found to have gapped edge states due to the too large staggered AB-sublattice potentials.

## Introduction

After successful preparation of graphene and discovery of its intriguing properties such as a massless Dirac fermion-like behavior of charge carriers at the Fermi level (*E*_*F*_) and the quantum spin Hall effect under spin-orbit interaction^1–3^, a great deal of attention has been paid to the two-dimensional (2D) materials of honeycomb structure^4–6^. Graphene, silicene, germanene and stannene are monatomic 2D materials of group IV elements^4,7^. Unlike the completely flat graphene layer, other monatomic 2D group IV materials consist of a buckled layer. Like graphene, monatomic 2D group IV materials are semimetals with linear band crossings at *E*_*F*_ and are quantum spin Hall insulators under spin-orbit interaction. Diatomic 2D materials consisting of group IV or group III-V elements, such as SiC and BN honeycomb sheets, are wide band gap semiconductors due to their ionic character possessing the potential to replace silicon in the semiconductor technology^5,6,8,9^.

Electrons in a two-dimensional honeycomb lattice can have a valley degree of freedom corresponding to the corners (K points) of the first Brillouin zone, in addition to the charge and spin degrees of freedom^10^. Like the charge and spin degrees of freedom, the valley degree of freedom is expected to open a new route to an advanced electronics. The valley degree of freedom can be distinguished in systems where inversion symmetry is broken^10–14^. Inversion symmetry breaking due to a staggered AB-sublattice potential removes the valley degeneracy and can give rise to a valley Hall effect where carriers in different valleys flow to opposite transverse edges. Under a small inversion symmetry-breaking potential, the Berry curvature is sharply peaked at each valley with opposite signs at opposite valleys, and the valley Chern number can be accurately defined^11,14^.

In a tight-binding (TB) model for 2D honeycomb lattice with a staggered AB-sublattice potential Δ, the Berry curvature is $\Omega (q)=3\Delta \xi /2{({\Delta }^{2}+3{q}^{2})}^{3/2}$ for small *q*, where *ξ*  =  ±1 is the valley index and *q* is the wavevector measured from the K point^11^. For small Δ, the Berry curvature is sharply peaked at the K point and the valley-resolved Chern number is given by *C*(*ξ*)  =  0.5*ξ* sign(Δ), which gives the valley Chern number *C*_*v*_  =  sign(Δ). As Δ increases, the Berry curvature peak broadens while maintaining its center at each valley, and the valley Chern number may deviate from the quantized value due to a partial mixing of the Berry curvature peaks pertaining to opposite valleys^15^. According to the bulk-edge correspondence, the number of gapless edge states leading to a quantum Hall effect should correspond to the bulk topology^16,17^. It is not clear whether the non-quantized valley Chern number resulting from large staggered AB-sublattiec potentials can correspond to gapless edge states.

In monatomic 2D group IV materials such as graphene, small staggered AB-sublattice potentials can arise from the substrate. In diatomic 2D honeycomb structures such as SiC and BN monolayers, large staggered AB-sublattice potentials are inherent due to their ionic character^5^. In a recent work on 2D honeycomb monolayers of SiC and BN, it was reported that inversion symmetry breaking opens a frequency gap in the phonon dispersion and a topologically protected interface phonon branch crossing over the frequency gap can be generated at a topological domain wall^18^. The valley-resolved Chern number for the lower boundary phonons of the frequency gap is 0.28*ξ*, which deviates from the ideal value of 0.5*ξ* and was attributed to a partial mixing of the Berry curvature peaks pertaining to the opposite valleys^15,18^.

Zigzag-edge graphene nanoribbon is a semimetal with nearly flat bands near *E*_*F*_ and has an insulating ground state with antiferromagnetic edge states under electron-electron interactions^19,20^. Under a transverse electric field, zigzag-edge graphene nanoribbon transforms to an antiferomagnetic half-metal^21^. While SiC and BN honeycomb monolayers are wide band gap insulators^5,6,8,9^, their zigzag-edge nanoribbons are metals with half-metallic ground states under electron-electron interactions^22–26^. For a quantum valley Hall insulator, the bulk-edge correspondence is known to be exactly established only at a topological domain wall^12^. 2D honeycomb lattice with a staggered AB-sublattice potential has topologically protected gapless edge states at a topological domain wall but not at the vacuum interface^11^. However, edge potentials at an edge can form a topological domain wall at the edge, and topologically protected gapless edge states can be formed even at the vacuum interface^11,27^. Nanoribbons inevitably have edge potentials, which can be induced by dangling *σ*-bonds, functional groups passivating the dangling bonds and edge-localized magnetic moments^27–29^. We wonder if the (half-)metallicity of the SiC and BN nanoribbons is due to the topologically protected gapless edge states corresponding to the quantum valley Hall effect.

In this work, we have investigated diatomic 2D honeycomb monolayers of SiC and BN by using the density functional theory (DFT) calculations. We found that the SiC monolayer has gapless edge states leading to a quantum valley Hall effect while the valley Chern number is not quantized. BN monolayer, however, was found to have gapped edge states due to the too large staggered AB-sublattice potentials. By using a TB model for 2D honeycomb lattice, we confirmed that gapless or gapped edge states exist for AB-sublattice potential difference less than or greater than the nearest-neighbor hopping energy, respectively.

**Figure 1:**
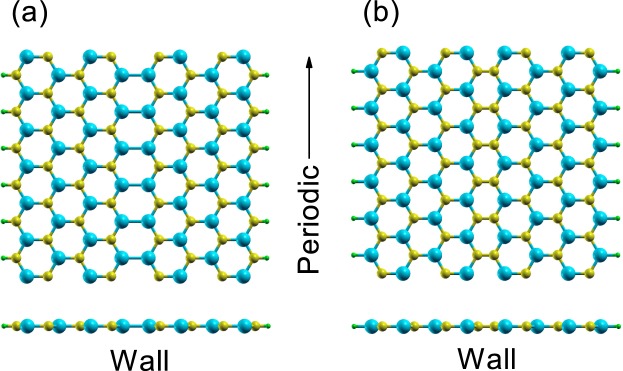
Geometric structures of H-terminated SiC 8-ZNR with a kink-type domain wall. Yellow, cyan and green balls correspond to carbon, silicon and hydrogen, respectively. (**a**) Both edges consist of carbon. (**b**) Both edges consist of silicon.

 Figure 1 shows the geometric structures of H-terminated zigzag-edge SiC nanoribbon with a kink-type domain wall in the middle of the nanoribbon. In Fig. 1(a), both edges consist of carbon atoms and the domain wall consists of Si-Si bonds, whereas in Fig. 1(b) both edges consist of silicon atoms and the domain wall consists of C-C bonds. In each domain, the staggered AB-sublattice potential Δ has the opposite sign. The structural domain wall can be a topological domain wall because the valley Chern number depends on the sign of Δ^11^.

**Figure 2:**
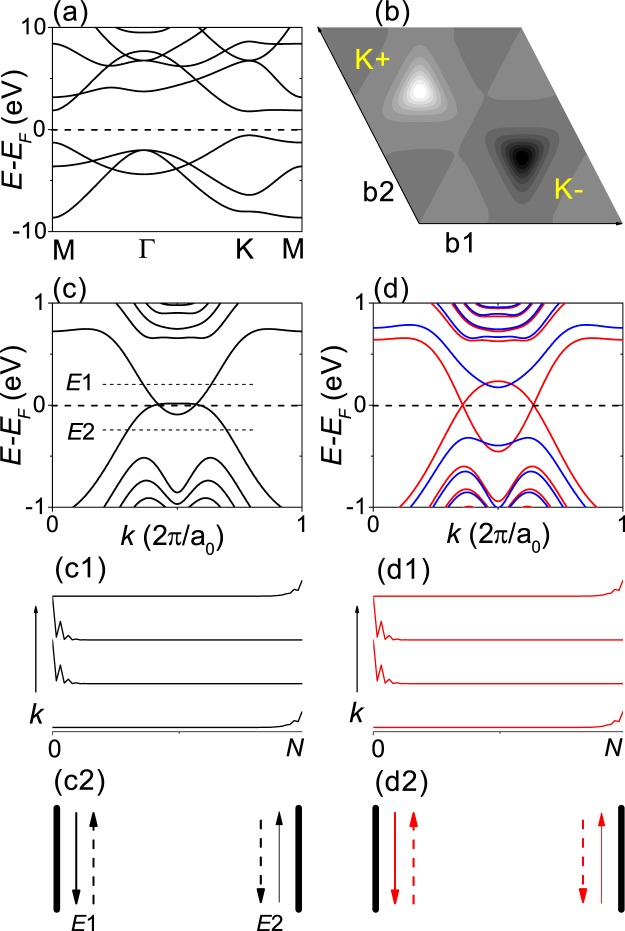
DFT calculations for SiC monolayer. (**a**) Bulk band structure. (**b**) Berry curvature map. The bright and dark spots correspond to positive and negative Berry curvature, respectively. b1 and b2 represent reciprocal lattice vectors. K+ and K− correspond to the opposite valleys. (c,c1,c2) and (d,d1,d2) Correspond to spin-restricted and spin-unrestricted calculations for H-terminated 32-ZNR, respectively. (**c,d**) show the band structures. (**c1,d1**) Show the square of the wavefunction ∣Ψ∣^2^ at *E*_*F*_. (**c2,d2**) Show schematics for the propagating states at *E*_*F*_. The red and blue colors represent the opposite spins. The up and down arrows correspond to the opposite propagating directions. The solid and dashed arrows represent the opposite valleys.

 Figure 2 shows the DFT calculations for SiC monolayer. In Fig. 2(a), the band structure indicates that SiC monolayer is a wide-gap insulator with a direct gap of about 2.4 eV at the K point. Figure 2(b) shows the Berry curvature map. The Berry curvature shows broad peaks of small height and has the opposite sign at the opposite valley, indicating a possible valley Hall effect. We obtained the valley-resolved Chern number *C*(*ξ*) ~ 0.18*ξ*, which gives the valley Chern number *C*_*v*_ ~ 0.36. The non-quantized valley Chern number may result from a partial mixing of the broad Berry curvature peak pertaining to each valley^15,18^ and we investigated zigzag-edge SiC nanoribbons in order to determine the corresponding valley Hall effect.

Figure 2(c,d) show the band structures obtained from the spin-restricted and spin-unrestricted calculations for H-terminated SiC 32-ZNR. The spin-unrestricted calculations led to a ferrimagnetic state^23^, where the spin polarization on the opposite edge is opposite in sign and different in magnitude. As shown in Fig. 2(c,c1), in the nonmagnetic state, there are gapless edge states within the bulk gap, which are well confined on an edge. Figure 2(c2) shows a schematic for the propagating states at *E*_*F*_. Since each state contributes *e*^2^/*h* to the conductivity, the Hall conductivity can be estimated in units of *e*^2^/*h* by counting the number of states propagating in a given direction. In Fig. 2(c2), we can obtain a quantized valley Hall conductivity *σ*_*v*_  =  2 *e*^2^/*h* and can confirm that backscattering of the gapless edge states is forbidden by the valley separation in the Brillouin zone because reversal of the propagating direction requires a reversal of the valley. When *E*_*F*_  =  *E*1 or *E*2 in Fig. 2(c), each corresponding to electron or hole doping, backscattering-free valley current flows along the opposite edge as indicated in Fig. 2(c2). *E*_*F*_ can be controlled by using gate devices and the backscattering-free valley current flowing on the opposite edge under doping of the opposite charge carrier can be used for experimental confirmation and application at room temperature.

As shown in Fig. 2(d,d1,d2), in the ferrimagnetic state, we can see that there are half-metallic edge states within the bulk gap, which are well confined on an edge and lead to a half-metallic quantum valley Hall effect with *σ*_*v*_  =  2 *e*^2^/*h*. In previous works, the half-metallic quantum valley Hall effect in a 2D honeycomb lattice with staggered AB-sublattice potentials and opposite spin polarization at opposite edges were explained by the topological confinement effect of the edge potentials^27^. The spin-dependent edge potentials with opposite signs at the opposite edges can form a topological domain wall only for a spin orientation and thus gapless edge states can be formed only for a spin orientation^27^. Because graphene and silicene are semimetals, their nanoribbons do not have gapless edge states and remain antiferromagnetic insulators^20,30^. No gapless edge state was found in H-terminated armchair-edge SiC nanoribbons as opposite valleys are completely mixed in armchair-edge nanoribbons.

**Figure 3:**
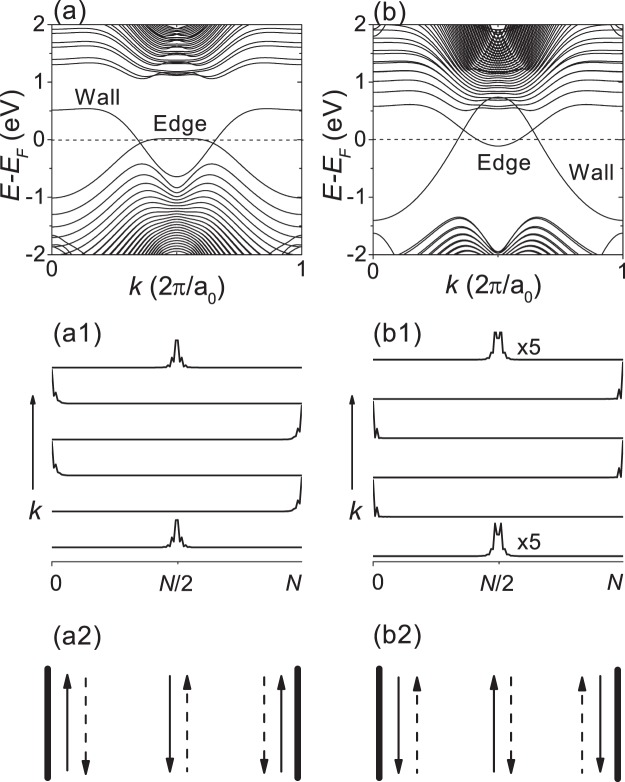
DFT calculations for H-terminated SiC 64-ZNR with a kink-type domain wall. (a,a1,a2) Both edges consist of carbon as shown in Fig. 1a. (b,b1,b2) Both edges consist of silicon as shown in Fig. 1b. (**a,b**) Show the band structures. (**a1,b1**) Show ∣Ψ∣^2^ at *E*_*F*_. (**a2,b2**) show schematics for the propagating states at *E*_*F*_. The up and down arrows correspond to the opposite propagating directions. The solid and dashed arrows represent the opposite valleys.

For more clarity, we investigated H-terminated SiC 64-ZNR with a kink-type domain wall in the middle of the nanoribbon, because the bulk-edge correspondence in a quantum valley Hall insulator is exactly established only at a topological domain wall^12^. Figure 3(a,a1,a2) correspond to the nanoribbon with both edges consisting of carbon as shown in Fig. 1(a), and Fig. 3(b,b1,b2) correspond to the nanoribbon with both edges consisting of silicon as shown in Fig. 1(b). We can see that there are gapless edge states well confined at the domain wall as well as at the vacuum interface, confirming that the two domains separated by the domain wall have different topology. According to the bulk-edge correspondence^12,13^, the number of gapless edge states per valley (per spin) at the topological domain wall should be equal to the difference of the valley-resolved Chern number between the two topological domains, *C*(*ξ*) − (−*C*(*ξ*))  =  2*C*(*ξ*). In Fig. 3(a2,b2), the number of gapless edge states per valley at the domain wall is 1 and *C*(*ξ*) should be 0.5, which indicates that the non-quantized valley Chern number obtained by integrating the Berry curvature is due to a partial mixing of the Berry curvature peaks pertaining to the opposite valleys.

**Figure 4:**
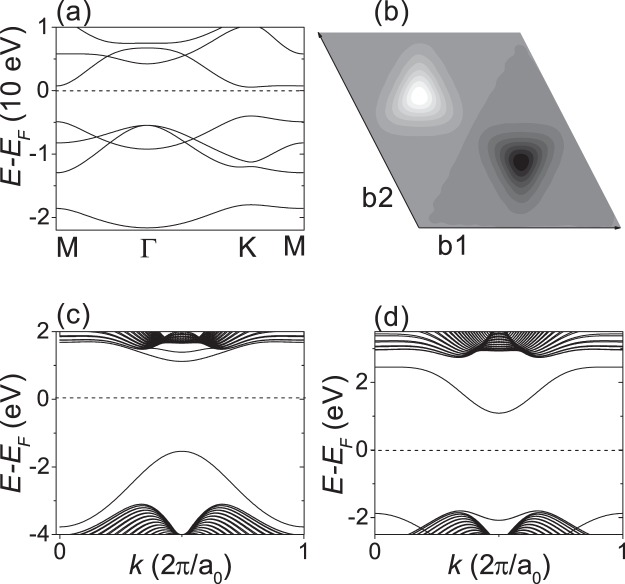
DFT calculations for BN monolayer. (**a**) Bulk band structure. (**b**) Berry curvature map. The bright and dark spots correspond to positive and negative Berry curvature, respectively. b1 and b2 represent reciprocal lattice vectors. (**c,d**) Show the band structures of H-terminated 64-ZNR with a kink type domain wall. (**c**) Both edges consist of boron. (**d**) Both edges consist of nitrogen.

 Figure 4 shows the DFT calculations for honeycomb BN monolayer. In Fig. 4(a), the band structure shows that BN monolayer is a wide-gap insulator with a direct gap of about 4.5 eV at the K point, which is much larger than that of SiC monolayer and can be attributed to a staggered AB-sublattice potential much larger than that of SiC monolayer. Figure 4(b) shows the Berry curvature map, which shows broad peaks of small heights and has the opposite sign at the opposite valley. The valley-resolved Chern number was *C*(*ξ*) ~ 0.18*ξ*, which is not quantized. H-terminated zigzag-edge nanoribbons were found to have no gapless edge states at the vacuum interface. Figure 4(c,d) show the band structures of H-terminated 64-ZNR with a kink-type domain wall. The band structures show gapped edge states, which do not fully cross the bulk gap. The gapped edge states can be attributed to the too large staggered AB-sublattice potentials.

**Figure 5:**
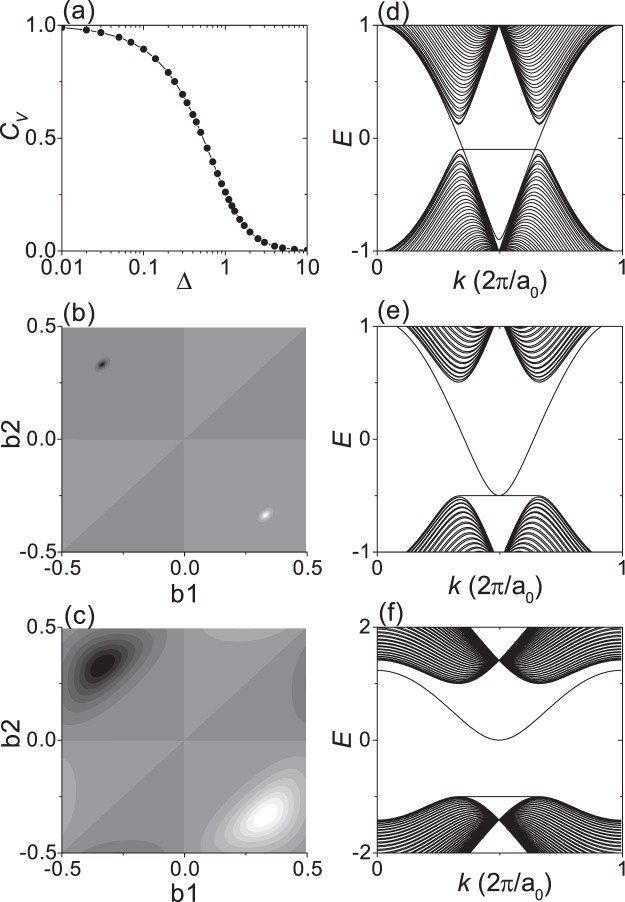
TB calculations for 2D honeycomb lattice with staggered AB-sublattice potentials. (**a**) Valley Chern number *C*_*v*_ as a function of Δ. (**b,c**) Show the Berry curvature maps for Δ = 0.1 and 1.0, respectively. (**d,e,f**) Show the band structures of 80-ZNR with a kink type domain wall, respectively, for Δ = 0.1, 0.5 and 1.0.

To clarify the effect of a large staggered AB-sublattice potential on the valley Chern number and gapless edge states, the TB model of Eq. (1) for a 2D honeycomb lattice was investigated. Figure 5 shows that the valley Chern number *C*_*V*_ continuously decreases with increasing staggered AB-sublattice potential Δ, indicating that *C*_*V*_ is not quantized except for the case of a very small Δ. As shown in Fig. 5(b,c), as Δ increases, the Berry curvature peaks pertaining to each valley are broadened and mixed with each other, giving a non-quantized valley Chern number. Figure 5(d–f) show the band structures of 80-ZNR with a kink type domain wall. We can see that there are gapless edge states for Δ less than *t*_*o*_/2 and gapped edge states for Δ greater than *t*_*o*_/2, each corresponding to SiC and BN monolayers.

In the TB mode, a topological phase transition from *C*_*v*_  =  +1 to *C*_*v*_  =  −1 occurs at Δ = 0, which has been well established for small Δ where *C*_*v*_ = sign(Δ)^11^, and the band gap linearly increases with ∣Δ∣. Therefore, according to the principle of adiabatic continuity, the TB model should be topologically equivalent regardless of ∣Δ∣ unless sign(Δ) changes. The TB model gives a quantum valley Hall phase for small Δ as is well known^10,11^, and the TB model should also give the quantum valley Hall phase for large Δ. The valley Chern number could be well defined only for very small Δ where each valley can be well isolated^11,14^. For large Δ, the existing definition of the valley Chern number does not seem to work well due to partial valley mixing. Just as the spin Chern number had to be redefined in consideration of spin mixing^31,32^, the valley Chern number needs to be redefined in consideration of the valley mixing, which requires further study.

**Figure 6:**
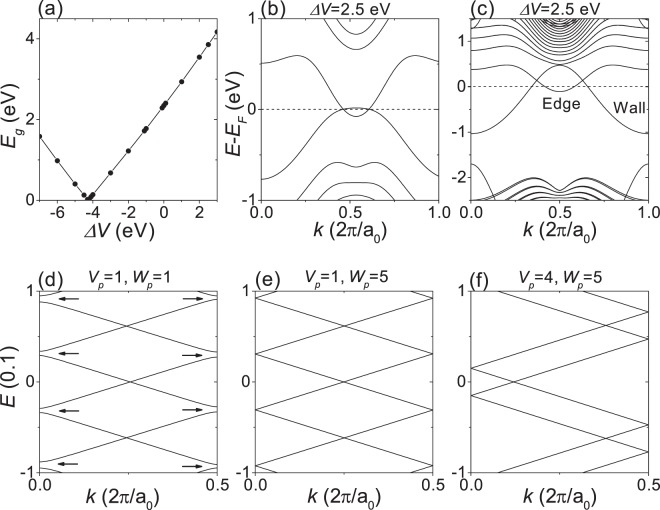
Topological phase transition and robustness of gapless edge states. (**a**–**c**) DFT calculations for SiC monolayer. (**a**) Band gap as a function of Δ*V*. (**b**) Band structure of H-terminated 32-ZNR when Δ*V* = 2.5 eV. (**c**) Band structure of H-terminated 64-ZNR with a kink-type domain wall whose edges consist of silicon when Δ*V* = 2.5 eV. (**d**–**f**) TB calculations for 20-ZNR with Δ_*eA*_  =  −Δ_*eB*_  =  Δ = 0.5 using a periodic supercell composed of 80 cells. Edge state bands within the bulk gap (**d**) when *V*_*p*_ = 1 and *V*_*w*_ = 1, (**e**) when *V*_*p*_ = 1 and *V*_*w*_ = 5, and (**f**) when *V*_*p*_ = 4 and *V*_*w*_ = 5. In (**d**), the small gaps indicated by arrows are a signature of backscattering.

The same is true for the SiC monolayer. Figure 6(a) shows the band gap of SiC monolayer as a function of Δ*V*, the applied potential difference between Si-3p and C-2p orbitals (see Methods section for details). The band structure of SiC monolayer is nearly insensitive to Δ*V* except for the band gap at the K point. The band gap closes at Δ*V*_*c*_ ~ −4.25 eV. *C*_*v*_ was calculated to be  +1 and  −1 just below and above Δ*V*_*c*_, respectively, indicating that the band gap closure corresponds to a topological phase transition from *C*_*v*_  =  +1 to *C*_*v*_  =  −1. The band gap linearly increases with ∣Δ*V* −Δ*V*_*c*_∣. Thus, we can consider that, unless the sign of (Δ*V* − Δ*V*_*c*_) changes, the SiC monolayer is in the topologically same phase regardless of the magnitude of (Δ*V* −Δ*V*_*c*_). When Δ*V* = 2.5 eV, the bulk band gap was about 3.86 eV, which is comparable to that calculated by the GWA method^8^. Figure 6(b,c) show the band structures of H-terminated 32-ZNR and of H-terminated 64-ZNR with a kink-type domain wall consisting of C-C bonds, respectively, when Δ*V* = 2.5 eV. We can see that the gapless edge states leading to a quantum valley Hall effect still exist within the bulk gap.

The robustness of the gapless edge states was verified by using the TB model of Eq. (1) for a large staggered AB-sublattice potential Δ = 0.5. We investigated 20-ZNR with Δ_*eA*_  =  − Δ_*eB*_  =  Δ using a periodic supercell composed of 80 cells. The edge potential Δ_*eA*_  =  − Δ_*eB*_  =  Δ forms a topological domain wall at each edge, ensuring the gapless edge states leading to a quantum valley Hall effect^11,27,29^. A single one-dimensional potential barrier centered at an edge site *i*_*p*_ was assumed on each edge, ${V}_{i}=({V}_{p}/{W}_{p}\sqrt{2\pi })$exp$[{(i-{i}_{p})}^{2}/2{W}_{p}^{2}]$, where *i* belongs to the edge sites. Figure 6(d–f) show some edge state bands within the bulk gap for three different potential barriers. As shown in Fig. 6(d), when *V*_*p*_  =  1 and *W*_*p*_ = 1, the small gaps at *k* = 0 and *k*  =  *π*∕*a*_0_ indicated by arrows are a signature of backscattering. When the barrier width *W*_*p*_ increases to *W*_*p*_ = 5 with *V*_*p*_ = 1 fixed, as shown in Fig. 6(e), all the energy gaps vanish, indicating that the backscattering disappears. Even when *V*_*p*_ increases to *V*_*p*_ = 4 with *W*_*p*_ = 5 fixed, there is no energy gap as shown in Fig. 6(f). Thus, the gapless edge states leading to a quantum valley Hall effect are robust against a smooth impurity scattering potential, which can be attributed to the large separation of the valleys in the Brillouin zone. For different edge potentials Δ_*eA*_  =  −Δ_*eB*_ = 0.2 and 0.8, we obtained essentially the same results.

To summarize, we have investigated wide-gap semiconductor SiC and BN monolayers by using the density functional theory calculations. The valley Chern number is not quantized in the diatomic 2D honeycomb monolayers, because the Berry curvature peaks pertaining to opposite valleys are broadened and partially mixed due to large staggered AB-sublattice potentials. Nevertheless, SiC monolayer has gapless edge states leading to a quantum valley Hall effect, and doping of the opposite charge carrier causes a backscattering-free valley current flowing on the opposite edge. On the other hand, BN monolayer has gapped edge states due to the too large staggered AB-sublattice potentials.

## Methods

A SIESTA package^33^, which uses a localized linear combination of numerical atomic-orbital basis sets, was employed for the DFT calculations. A generalized gradient approximation of Perdew-Burke-Ernzerhof was used for the exchange and correlation potential^34^. A double-*ζ* polarized basis set was used and norm-conserving Troullier-Martins pseudopotentials generated with a Perdew-Burke-Ernzerhof functional were used. The plane-wave cutoff energy of 350 Ry was used for the real-space grid. *k*-points of 100 × 100 × 1 and 50 × 1 × 1 meshes in a Monkhorst-Pack scheme were used for 2D sheets and nanoribbons, respectively. The atomic coordinates were optimized by using the conjugated gradients method with a maximum force tolerance of 0.1 eV/nm. The equilibrium lattice constant *a*_0_ = 0.311 nm and 0.251 nm, respectively, for SiC and BN monolayers were obtained from total energy minimum, in agreement with previous works^5^. Zigzag-edge nanoribbons were considered as a one-dimensional system periodic in a zigzag direction and are referred to as *N*-ZNR, where the ribbon width is represented by the number *N* of the Si-C (B-N) pairs in the unit cell. The nanoribbon edge was terminated with hydrogen to remove the dangling *σ*-bonds. The spin-orbit coupling, much smaller than the band gap, was neglected. If not specified, we deal with spin-restricted calculations.

To apply a staggered AB-sublattice potential, the LDA + U method implemented in SIESTA was used with the option that the U parameter is interpreted as a local potential shift. The applied potential difference between Si-3p and C-2p orbitals corresponds to Δ*V*  =  *U*_*Si*−3*p*_ − *U*_*C*−2*p*_, where *U*_*Si*−3*p*_ and *U*_*C*−2*p*_ are the U parameters for Si-3p and C-2p orbitals, respectively.

Using the wavefunctions obtained from the DFT calculations, maximally localized Wannier functions were constructed within the Wannier90 code^35^. Berry curvatures were calculated based on the Wannier interpolation. Using the anomalous Hall conductivity calculation routine implemented in Wannier90 code, the valley-resolved Chern number *C*(*ξ*) was calculated by integrating the Berry curvature over a half Brillouin zone. The valley Chern number was obtained as *C*_*v*_  =  *C*(K+) − *C*(K−) according to previous works^36^.

In diatomic 2D honeycomb structures such as SiC and BN, *p*_*z*_ orbital electrons feel different on-site potentials at different sublattices due to charge transfer between the two kinds of atoms, and can be modeled by a *π*-band TB model with staggered AB−sublattice potentials^36,37^:

1 $$H=-{t}_{0}\sum _{\left\langle i,j\right\rangle }{c}_{i}^{\dagger }{c}_{j}+\sum _{i}{\Delta }_{i}{c}_{i}^{\dagger }{c}_{i}.$$

*t*_0_ = 1 is the nearest-neighbor hopping energy. The staggered AB-sublattice potential Δ_*i*_ was set to Δ_*A*_  =  −Δ for the A-sublattice and Δ_*B*_ = +Δ for the B-subalttice. In zigzag-edge nanoribbons, each edge consists of only A- or B-sublattices. Edge potentials can be included by letting Δ_*i*_  =  Δ_*eA*_ for an edge site *i* belonging to the A-sublattice and Δ_*i*_ = Δ_*eB*_ for an edge site *i* belonging to the B-sublattice. Edge potentials can form a topological domain wall at each edge if sign(Δ_*eA*_) = sign(Δ_*B*_) and sign(Δ_*eB*_) = sign(Δ_*A*_)^27,29^.
